# Examining the Appeal of Cigarette Filters: An Online Randomised Experiment Among Adults in Great Britain

**DOI:** 10.64898/2026.09.17.26363313

**Published:** 2026-09-20

**Authors:** Katherine East, Hazel Cheeseman, Eve V Taylor

## Abstract

Background and aims. Cigarette filters are present in almost all cigarettes sold globally, despite providing no health protection and contributing substantially to environmental pollution. Filters may also increase cigarette appeal and reinforce misperceptions of reduced harm. This study aimed to examine the impact of cigarette filters on cigarette appeal and perceptions of health and environmental harms among adults in Great Britain. Design. Online between-subject randomised experiment embedded within a nationally representative survey. Setting. Great Britain, May 2026. Participants. Adults aged 18+ years, recruited through the YouGov Social Omnibus survey (n=3961). Subgroup analyses were conducted among people who currently smoked (n=499) and people who smoked daily (n=270). Intervention and comparator. Participants were randomised to view images of cigarettes either without filters (intervention) or with filters (conventional cigarettes; comparator). Measurements. The primary outcome was selecting one of the displayed cigarettes to smoke vs. selecting none. Secondary outcomes were perceptions that the cigarette was extremely harmful to health and extremely harmful to the environment. Logistic regression models were used, adjusting for age, sex and smoking status. Results. There was strong evidence to suggest that participants who viewed cigarettes without filters had markedly lower odds of choosing a cigarette to smoke than those who viewed cigarettes with filters (19.1% vs. 28.1%; adjusted odds ratio [AOR]=0.46 [95% confidence interval [CI]=0.38-0.55], p<.001). Cigarettes without filters also had higher odds of being perceived as extremely harmful to health (65.2% vs. 59.9%; AOR=1.26 [1.10-1.45], p=.001). Evidence was insufficient to conclude a difference between conditions in perceiving cigarettes as extremely harmful to the environment (41.8% vs. 44.3%; AOR=0.89 [0.78-1.01], p=.074). Findings were similar among people who currently smoke, with participants who viewed cigarettes without (vs. with) filters having markedly lower odds of choosing a cigarette (67.4% vs. 94.2%; AOR=0.12 [0.06-0.22], p<.001). These findings were also similar among people who smoke daily (70.7% vs. 94.8%; AOR=0.14, 95% CI=0.06-0.36, p<0.001). However, evidence was insufficient to conclude differences in perceived health harms between conditions among people who smoke currently (29.7% vs. 27.8%; p=.588) or daily (32.2% vs. 28.6%; p=.324). Summary. Removing cigarette filters reduced the appeal of cigarettes among the general population and people who smoke. Removing filters also increased perceptions of health harms in the general population. Given that cigarette filters provide no health protection and contribute to environmental pollution, policies that regulate or prohibit filters may support public health and environmental goals.

## Full Text Availability

The license terms selected by the author(s) for this preprint version do not permit archiving in PMC. The full text is available from the preprint server.

